# The Role of Autophagy in Breast Cancer Metastasis

**DOI:** 10.3390/biomedicines11020618

**Published:** 2023-02-18

**Authors:** Hye Min Kim, Ja Seung Koo

**Affiliations:** 1 Department of Pathology, Yongin Severance Hospital, Yonsei University College of Medicine, Yongin 16995, Republic of Korea; 2 Department of Pathology, Yonsei University College of Medicine, Seoul 03722, Republic of Korea

**Keywords:** autophagy, breast cancer, metastasis, autophagy modulators

## Abstract

Patient morbidity and mortality is significantly increased in metastatic breast cancer. The metastasis process of breast cancer is very complicated and is delicately controlled by various factors. Autophagy is one of the important regulatory factors affecting metastasis in breast cancer by engaging in cell mobility, metabolic adaptation, tumor dormancy, and cancer stem cells. Here, we discuss the effects of autophagy on metastasis in breast cancer and assess the potential use of autophagy modulators for metastasis treatment.

## 1. Introduction

Autophagy is defined as the lysosomal degradation of a cellular component. It is divided into three different types: microautophagy, chaperone-mediated autophagy, and macroautophagy. Macroautophagy is the major type and is usually simply referred to as autophagy. Generally, autophagy performs an important homeostatic role by removing dysfunctional or damaged cellular components to recycle essential cellular components [1,2,3,4]. Autophagy is considered to exert an important role in normal physiologic conditions, as well as in the process of tumor biology. It inhibits the accumulation of genetic and genomic defects that could induce the occurrence of neoplasm; therefore, it acts as a tumor suppressor. However, the role of autophagy as a tumor promoter is also recognized. Cancer cells generally survive through angiogenesis and/or aerobic glycolysis in hypoxic environments with decreased nutrients. Meanwhile, these compensations may be insufficient for highly aggressive malignant tumors that require high metabolic demand. In this case, energy is supplied to cells by recycling cytoplasmic components via autophagy as an alternative metabolic pathway [5,6]. Meanwhile, unrestricted autophagy leads to progressive loss of cellular components, inducing autophagy-related cell death eventually [7,8].

Breast cancer is the most common malignancy in women, showing an aggressive feature. The mortality in breast cancer is attributed to distant metastasis that occurs in the brain, bone, liver, and the lung in nearly 90% of cases; metastasis is composed of a multicellular and multistep process [9,10]. Among patients with breast cancer, approximately 30% of them are reported to distant metastasis, having a median survival time of 2 years; the 5-year survival rate is estimated to be 30% when metastasis to a distant organ is present [11,12,13,14]. Various studies have been conducted in recent years to elucidate the role of autophagy in leveraging metastasis of breast cancer. This review summarizes the role of autophagy in breast cancer metastasis. Furthermore, the potential of autophagy modulators as therapeutic targets for breast cancer metastasis is discussed.

## 2. General Aspects of the Autophagy Process

Autophagy is a mechanism that degrades intracellular components and recycles proteins and intracytoplasmic organelles to produce amino acids, nucleotides, fatty acids, sugars, and adenosine triphosphate, which are necessary for cell survival. It is activated in stressful situations such as hypoxia and nutrient deprivation [15,16]. The autophagy process is largely divided into a canonical pathway and a non-canonical pathway. The canonical pathway, which generally refers to a general autophagy process, is characterized by a systematic recruitment of autophagy-related (ATG) proteins to form a phagophore. On the other hand, the formation of an autophagosome without these key ATG proteins comprises a non-canonical pathway [17]. The process of the canonical autophagy pathway can be broadly divided into five stages; the procedure of each stage and the components involved are as follows: The first step is initiation, including stressful circumstances such as low energy, reactive oxygen species (ROS), and nutrient deprivation inhibit mammalian target of rapamycin (mTOR) and activate Unc-51-like kinase 1 (ULK1) [18]. The second step is the nucleation/elongation step, in which activated ULK1 recruits and activates the ATG9-ATG2 complex and the Beclin1 complex to form a phagophore. At this stage, the Beclin1 complex induces membrane elongation, using an ubiquitin-like conjugation system such as ATG5, ATG12, or ATG16. In the third step, maturation occurs after forming an ubiquitin-like conjugation system including light chain 3 (LC3) and ATG8 [19]. Subsequently, by conjugation with ATG4 protease and phosphatidylethanolamine (PE), LC3-1, gamma-aminobutyric acid receptor-associated protein (GABARAP)-I is converted into LC3-II, GABARAP-II and is integrated into an isolation membrane via ATG5/12/16L and ATG3/7 complexes to eventually form an autophagosome [20]. The fourth refers to a fusion step, where autophagosome and lysosome fuse, mediated by Rab7 and soluble N-ethylmaleimide-sensitive factor attached protein receptor (SNARE) [15]. The final step is the degradation step, where the intracellular components and organelles within the autolysosome—formed by the fusion of autophagosome and lysosome—are degraded through hydrolysis by lysosomal protease to form building blocks such as amino acids and are used for recycling. Several proteins are involved in the process. Beclin1 and its binding partner class III phosphoinositide 3-kinase (PI3KCIII/ also known as Vps34) help initiate formation of the autophagosome [21]. Two ubiquitin-like conjugation systems are involved in autophagosome elongation and completion [22]: one forms the ATG12-ATG5/ATG16 complex and the other forms the PE-conjugated ATG8, a representative mammalian ortholog of ATG8, a LC3 [23]. In addition, autophagy receptors bind to ubiquitinated proteins and deliver them to the autophagosome: p62, neighbor of BRCA1 gene 1 (NBR1), optineurin (OPTN), NDP52, B-cell lymphoma (Bcl)-2 interacting protein 3 (BNIP3), Tax1-binding protein 1 (TAX1BP1), and nuclear receptor coactivator-4 (NCOA4) [24,25]. These autophagy receptors are not degraded by autophagy. Inhibition of autophagy induces cytosolic accumulation, with p62 and NBR1 acting as important signaling scaffolds [26,27]. Unlike the canonical pathway, in which ATG proteins and non-ATG proteins are systematically involved in autophagosome formation, autophagosomes are formed by bypassing some steps and/or proteins in the non-canonical pathway. Examples of these cases include when autophagy-related 7 (ATG7), ATG5, and LC3 are bypassed in the elongation step, ULK1 is bypassed in the initiation step, and BECLIN1 is bypassed in the nucleation step [17]. Autophagy is dependent on the ULK1 complex in Beclin1-independent autophagy [28]. The ULK1 complex, which is composed of ULK1, ATG13, ATG101, and 200 kDa FAK-family interacting protein (FIP200), moves to the autophagy initiation site and activates vacuolar protein sorting (Vps)34 complex (class III phosphatidylinositol 3-kinase VPS34, BECLIN1, VPS15, and ATG14L (ATG14-like)) and influences ATG9 trafficking to induce phagophore formation [29]. The ULK1 complex may be either activated or suppressed by phosphorylation, acetylation, and ubiquitylation; the representative enzymes that modify ULK1 complex are mTOR [30], adenosine monophosphate-activated protein kinase (AMPK) [30,31], 60-kDa Tat-interactive protein (TIP60) [32], TNF receptor-associated factor (TRAF6) [33], and mitochondrial E3 ubiquitin-protein ligase 1 (MUL1) [34]. In the case of the mTOR pathway, the ULK1 complex is integrated with mTOR complex 1; when autophagy is initiated, mTOR complex 1 dissociates with the ULK1 complex and dephosphorylation of the inhibitory site and autophosphorylation of the ULK1-activating site occurs [35]. Activation of ULK1 results in the phosphorylation of ATG13 and FIP200 and the activated ULK1 complex is translocated from the cytosol into the endoplasmic reticulum [36].

## 3. The Role of Autophagy in Breast Cancer Metastasis

Autophagy generally affects normal cells under physiologic status together with tumor cells. The proposed mechanism of autophagy in suppressing tumors includes oncogenic protein degradation, induction of oncogene-induced senescence, anti-inflammatory function, preservation of genetic stability, maintenance of normal stem cells, an antiviral/antibactericidal effect, and maintenance of normal metabolism [37,38,39]. In contrast, autophagy may promote tumorigenesis by influencing the resistance to hypoxia and anoikis, resistance to starvation, survival of senescent cancer cells, maintenance of cancer stem cells, and resistance to therapy-induced cell death [37,38,39]. Similar to different cancers, autophagy has a diverse effect in breast cancer and may exert tumor-promoting and tumor-suppressing effects in each step of tumorigenesis. The general effect of autophagy in breast cancer [40,41,42] is not the main focus of this study, however we have briefly summarized it below. First, the tumor-promoting role of autophagy in the tumor-initiating stage of breast cancer is the function of promoting breast cancer stem cells (BCSCs). Possible mechanisms include maintaining BCSC homeostasis, increasing BCSC markers’ clusters of differentiation (CD)44 and aldehyde dehydrogenase (ALDH), upregulating the epidermal growth factor receptor (EGFR)/ signal transducer and activator of transcription (STAT)3 pathway, interleukin (IL)-6/STAT3 pathway, transforming growth factor (TGF)/ suppressor of mothers against decapentaplegic homolog (SMAD) pathway, and maintaining BCSC lysosomal integrity [43]. Meanwhile, the tumor-suppressing role of autophagy includes inhibiting autophagy-related BCSC death and DNA damage. The tumor-promoting role of autophagy in breast cancer carcinogenesis and cell proliferation includes increased glycolysis, increased cyclin D1 expression, RAS pathway, mitogen-activated protein kinase (MAPK)/ nuclear factor erythroid 2-related factor 2 (NRF2) pathway, Src family of protein tyrosine kinases (SRC) pathway, and integrin β1 signaling [44]. In contrast, the role of autophagy in tumor suppression includes maintaining cellular homeostasis (which is also relevant to human epidermal growth factor receptor 2 (HER-2) pathway inhibition), Wnt family member 1 (WNT1) pathway inhibition, Bcl2 inhibition, neurogenic locus notch homolog protein 1 (Notch1) degradation, and forkhead box O3 (FOXO3) signaling [45]. Autophagy is involved in the stages of tumor initiation, tumorigenesis, and proliferation in breast cancer and also in the metastatic process. It acts both as a prometastatic and antimetastatic factor depending on the stages; these mechanisms are described below.

### 3.1. Regulator for Migration, Invasion, and Anoikis

**Figure 1:**
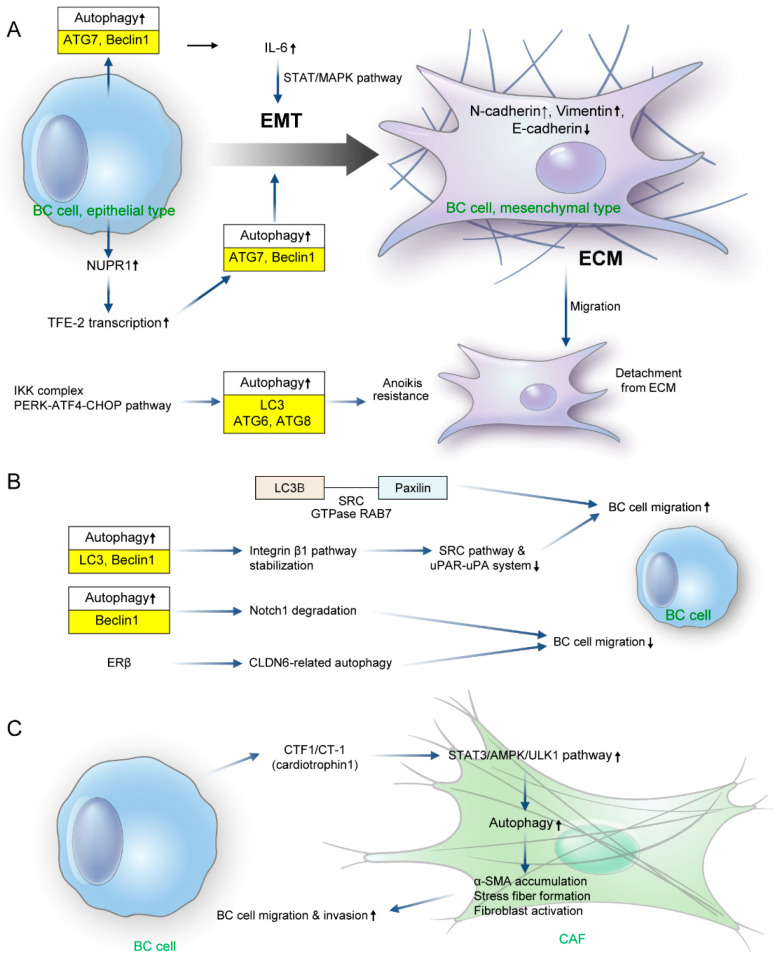
The role of canonical autophagy pathway for migration and invasion in breast cancer metastasis. (**A**) Epithelial–mesenchymal transition regulator. Autophagy promotes the epithelial–mesenchymal transition (EMT) in breast cancer. Activated autophagy in breast cancer cells activates EMT through increased TFE-2 transcription (and upregulation of NUPR1) and the STAT/MAPK pathway involving IL-6. Autophagy markers related to IL-6 are autophagy markers ATG7 and Beclin1 and TFE-2 related autophagy markers are LC3, Beclin1, and ATG5. During this process, the expression of N-cadherin and vimentin increases, while that of E-cadherin decreases. Cell death caused by anoikis should occur following cancer cell migration after detachment from the extracellular matrix (ECM). Nevertheless, autophagy is involved in inducing anoikis resistance of breast cancer cells through the IKK complex and the PERK-ATF4-CHOP pathway. IKK complex activates LC3, whereas ATG6 and ATG8 are activated by the PERK-ATF4-CHOP pathway. (**B**) Interaction of breast cancer cells to ECM-related proteins. Paxillin is one of the adhesion proteins that interacts with LC3B. This increases breast cancer cell migration through SRC or Rab7 GTPase. Furthermore, autophagy through LC3 and Beclin1 increases breast cancer cell migration by stabilizing the integrin β1 pathway and inhibiting the SRC pathway and the uPAR-uPA system. However, autophagy through Beclin1 also suggests a mechanism for suppressing migration and invasion of breast cancer cells through Notch1 degradation or CLDN6-related autophagy by ERβ. (**C**) Crosstalk with CAF. STAT3/AMPK/ULK1 activation occurs in CAFs by cardiotrophin 1 (CTF1/CT-1) secreted from breast cancer cells. This induces α-SMA accumulation, stress fiber formation, and fibroblast activation, which eventually increases breast cancer cell migration and invasion. BC—breast cancer; NUPR1—nuclear protein 1; TFE-2—transcription factor E-2; IL-6—interleukin-6; STAT—signal transducer and activator of transcription; MAPK—mitogen-activated protein kinase; EMT—epithelial–mesenchymal transition; ECM—extracellular matrix; IKK—IκB kinase; PERK—protein kinase R–like endoplasmic reticulum kinase; ATF4—activating transcription factor 4; CHOP—C/EBP homology protein; LC3—light chain 3; SRC—Src family of protein tyrosine kinases; GTP—guanosine tri-phosphate; uPAR—urokinase-type plasminogen activator receptor; uPA—urokinase-type plasminogen activator; Erβ—estrogen receptor beta; CLDN6—claudin 6; AMPK—adenosine monophosphate-activated protein kinase; ULK1—unc-51-like kinase 1; SMA—smooth muscle actin; CAF—cancer-associated fibroblast; ATG—autophagy related.

The metastatic process of breast cancer can be divided into two stages: the physical translocation of cancer cells from a primary to a distant site and the colonization at the metastatic site. Physical translocation is the process of tumor dissemination wherein tumor cells spread throughout the body; the most important and fundamental process is tumor cell invasion and migration [46,47,48]. Epithelial–mesenchymal transition (EMT) is an important process during tumor metastatic dissemination of solid tumors, including breast cancer. The basic concept of EMT is that epithelial cells are morphologically and functionally converted into mesenchymal cells leading to various events: (i) cell shape changes from round to ovoid or spindle, (ii) phenotypic changes of cell markers, (iii) loss of cell junction protein (E-cadherin, tight junction, desmosome, and integrin) and cytoskeletal elements including cytokeratin, and expression (gain) of mesenchymal cadherin (N-cadherin and cadherin-11), vimentin-rich intermediate filament, and motility associated actin [49]. Extracellular stimuli that trigger EMT include Hedgehog, epidermal growth factor (EGF), hepatocyte growth factor (HGF), TGF-β, Wnt, fibroblast growth factor (FGF), and insulin-like growth factor (IGF), while transcriptional factors that act as drivers include SNAIL1, SNAIL2 (SLUG), SIP-1 (ZEB-3), ZEB-1 (δEF1), and TWIST [50]. An important regulator of EMT in breast cancer is IL-6; it is highly expressed in adipocytes and is abundant in normal breast tissue. IL-6 activates the Janus kinase/STAT pathway and the MAPK pathway, reduces the expression of E-cadherin, and promotes EMT by inducing the expression of TWIST, SNAIL, N-cadherin, and matrix metalloproteinase 9. IL-6 secretion is tightly regulated by autophagy, and the autophagy activity in breast cancer cells and tumor microenvironment cells regulates EMT mediated by the IL-6/STAT and IL-6/MAPK pathways [51,52,53,54]. In fact, autophagy in RAS-transformed mammary cancer cells induces tumor cell invasion and lung metastasis through multiple secretory factors including IL-6 [55]. Nuclear protein 1 (NUPR1), which is associated with metastasis in mice, is upregulated in breast cancer cells and tissues. Stimulation of autophagy through transcription factor E3 transcription activation by NUPR1 results in an increase in the expression of EMT-related molecules N-cadherin and vimentin and a decrease in the expression of E-cadherin [56]. The regulation of breast cancer EMT by autophagy differs according to disease subtypes. Autophagy induction of cell migration and invasion following the nuclear translocation of Yes-associated protein (an oncogenic effector of the Hippo signaling pathway) appears in triple negative breast cancer (TNBC) and not in estrogen receptor (ER)-positive breast cancer [57]. Ectopic expression of death effector domain-containing DNA-binding protein (DEDD) stabilizes BECLIN1 and VPS34 through physical interaction in a TNBC subtype cell line (MDA-MB-2310). This stabilization causes SNAIL and TWIST degradation through autophagy, impeding the EMT process. Meanwhile, DEDD inactivates and degrades VPS34 in the ER-positive breast cancer (luminal type) cell line of MCF-7, which inhibits autophagy and triggers SNAIL and TWIST accumulation to promote EMT [58]. Tumor cells migrate by EMT and are detached from the surrounding extracellular matrix (ECM). Meanwhile, anoikis is a type of programmed cell death involving the detachment of normal anchorage-dependent cells from the surrounding ECM [59]. Autophagy is involved in the process of anoikis. A three-dimensional culture study using normal mammary epithelial cells shows that autophagy regulates spontaneous apoptosis when the ECM detaches during cell formation of acini [60]. The IκB kinase (IKK) complex and protein kinase PKR-like ER kinase (PERK)-eukaryotic translation initiation factor 2α (eIF2α)-activating transcription factor 4-C/EBP homology protein (CHOP) pathway inhibits anoikis by autophagy induction during ECM detachment in mammary epithelial cells [60,61]. Furthermore, the levels of luminal P-PERK and LC3 increase in ductal carcinoma tissues in situ compared with in normal breast tissues [61]. Prosurvival autophagy in the process of ECM detachment in breast cancer is oncogene specific and involves a phosphatidylinositol 4,5-bisphosphate 3-kinase catalytic subunit alpha mutation but not an HRAS mutation [55,60]. One of the key processes of metastasis is the interaction of tumor cells with ECM components. Complex proteins connected to the ECM called focal adhesions (FAs) play a role as scaffolds during the migration of metastatic cancer cells. The components of FA include vinculin, integrin, paxillin, and focal adhesion kinase, which act on cell signaling to regulate various cell biology [62,63]. Autophagy is involved in FA disassembly during the dismantling of migrating cells as autophagosomes formed from autophagy colocalize with FAs. Suppression of autophagy through ATG7/12 silencing in breast cancer cells increases FA and decreases the breast cancer cell migration rate. This suggests that autophagy acts on FA turnover to promote migration and invasion. Paxillin is an adhesion protein that interacts with LC3B and LC3 interacting region (LIR) domains. Inhibition of autophagy by ATG5 or ATG7 silencing results in paxillin accumulation and increased cell motility. Tyrosine 40 in the LIR domain of paxillin is a target of SRC kinase; SRC increases the LC3B–paxillin interaction and promotes the migration of breast cancer cells [64,65]. Rab7 is a GTPase that contributes to autophagosome–lysosomal fusion, phosphorylates paxillin tyrosine 118, and increases paxillin turnover through autophagy [66]. In addition, the integrin β1 pathway is involved in breast cancer cell migration and invasion by autophagy. It inhibits the SRC pathway and the urokinase-type plasminogen activator receptor urokinase plasminogen activator system, which play an important role in cell migration and invasion. Inhibition of autophagy through microtubule-associated protein 1 light chain 3 beta (MAP1LC3) or B silencing destabilizes the integrin β1 pathway [67]. Autophagy inhibits the migration of breast cancer cells. Autophagy inhibition by Beclin1 silencing inhibits Notch1 degradation, increases breast cancer cell migration [68] and ERβ (known to act as a tumor suppressor in breast cancer), transactivates claudin-6 (CLDN6), and induces CLDN6-related autophagy to suppress migration and invasion in breast cancer [69]. Interactions between breast cancer and cells that consist of the tumor microenvironment (especially cancer-associated fibroblasts (CAFs)) play an important role in tumor invasion and metastasis. Autophagy is also involved in this process. Cardiotrophin 1 (CTF1/CT-1) is a cancer cell-derived secreted factor that is an autophagy activator of CAF [70]. CTF1 activates the key regulators of autophagy (STAT3, AMPK, and ULK1), which results in CTF1-dependent ACTA2/alpha-smooth muscle actin accumulation, stress fiber formation, and fibroblast activation. CTF1 expression is increased in breast cancer tissues and is associated with lymph node metastasis [70]. This indicates that CTF1 and autophagy are involved in breast cancer cell migration and invasion by interaction with CAF (Figure 1).

### 3.2. Modulator for Tumor Dormancy

Some cases of breast cancer metastasis are initially diagnosed with distant metastasis, but the majority of cases experience distant metastasis several years after initial treatment and approximately 90% of deaths from breast cancer are attributed to this. One reason for this phenomenon is that dormant breast cancer cells are reactivated [71,72]. Tumor dormancy is a mechanism for disseminated tumor cells to adapt to an unfavorable microenvironment, resulting in complete withdrawal from the cell cycle [73]. In the early stage of breast cancer, tumor cells can be disseminated throughout the body but remain in a dormant status for various reasons. A change in the tumor environment by a specific signal (such as autophagy) can convert tumor cells to proliferative status [73,74,75,76,77]. The upregulation of autophagy in the dormant stage in breast cancer cells rather than in the proliferative stage indicates that autophagy facilitates tumor dormancy [71]. Breast cancer cells activate the PI3K system under unfavorable conditions. This leads to inhibition of the AKT/mTOR pathways, results in autophagy activation, and causes tumor cells to enter dormancy. Examination of dormant breast cancer cells revealed that the expression of the tumor suppressor gene DIRAS3/ARHI (which suppresses the AKT-mTOR pathway and activates autophagy) was high [74,78,79]. Chemotherapy of breast cancer initiates ectopic expression of DIRAS3/ARHI, inducing autophagy and initiating tumor dormancy [78,79]. Therefore, inhibition of autophagy in breast cancer is expected to cause a prometastatic effect. Stable autophagy inhibition via ATG5 knockdown resulted in dormancy escape and earlier metastatic recurrence in adriamycin-induced dormant breast cancer cells compared with wild-type cells [80]. In addition, genetic inhibition of autophagy in breast cancer increases metastasis to the lung and disseminated tumor cells cause overgrowth to overt macrometastasis; this is explained by the intracellular deposition of autophagy receptor, NBR1 [81,82]. Intracellular accumulation of NBR1 is also involved in prometastatic differentiation; it induces aggressive basal differentiation through the expression of cytokeratin 14 and TP63 in breast cancer. Intracellular accumulation of NBR1 is also involved in prometastatic differentiation that induces aggressive basal differentiation such as cytokeratin 14 and TP63 in breast cancer [81,82]. Inhibition of autophagy in dormant metastatic breast cancer cells by silencing ATG7 or administering hydroxychloroquine (HCQ; an autophagy inhibitor) induces mitochondrial dysfunction by reducing mitophagy; this elevates ROS levels and reduces dormant tumor cell survival, thereby suppressing a switch from dormant status to proliferation status [71]. Dormant breast cancer cells show low levels of 6-phosphofructo-2-kinase/fructose-2,6-biphosphatase 3 (PFKFB3) and high autophagy activity, whereas metastatic breast cancer cells show high levels of PFKFB3 and low levels of autophagy. PFKFB3 produces fructose 2,6-bisphosphate to regulate glycolysis and directly interacts with autophagy receptor (p62) to regulate autophagosome degradation. A BCSC transcriptome analysis of breast cancer showed high levels of PFKFB3, which is associated with metastatic activity in high-grade cancers (including TNBC and HER-2-enriched breast cancer). Therefore, it can be proposed that low-level autophagy causes PFKFB3 stabilization to cause a switch from dormant tumor to active metastatic tumor cells. Indeed, autophagy-related protein silencing in dormant breast cancer cells stabilize PFKFB3 [83]. Reduction of mitogenic signals by impaired integrin and growth factor signaling induces tumor dormancy in breast cancer [84,85]. Inhibition of the integrin β1 pathway induces autophagy in ECM-detached cells [86], with disruption of integrin β1-mediated autophagy induction leading to tumor dormancy.

### 3.3. Cancer Stem Cell Regulator

**Figure 2:**
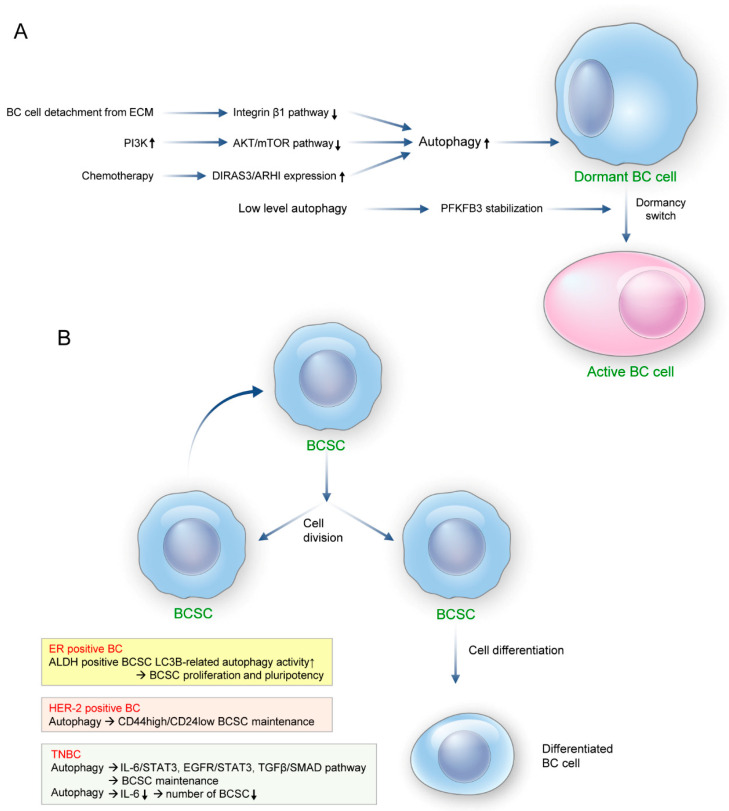
The role of autophagy in tumor dormancy and cancer stem cell biology in breast cancer metastasis. (**A**) Tumor dormancy regulator. Activation of autophagy in breast cancer cells promotes tumor dormancy. The mechanisms of autophagy activation include inhibition of the integrin β1 pathway by breast cancer cells detached from the ECM detachment, activation of the PI3K system in breast cancer cells, inhibition of the AKT and mTOR pathways, and DIRAS3/ARHI ectopic expression by chemotherapy. Low-level autophagy in breast cancer cells causes PFKFB3 stabilization, resulting in a switch from dormant to active metastatic tumor cells. (**B**) Modulator for cancer stem cell maintenance. In breast cancer, autophagy involved in the maintenance of breast cancer stem cells (BCSCs) shows differences according to molecular subtype. LC3B-related autophagy activity was high (and involved BCSC proliferation and pluripotency) in ALDH1-positive stem cells derived from ER-positive breast cancer. Meanwhile, autophagy was involved in CD44high/CD24low BCSC maintenance in HER-enriched breast cancer. Autophagy is involved in BCSC maintenance in TNBC through the IL6/STAT3, EGFR/STAT3, and TGFβ/SMAD pathways. This suggests that autophagy may reduce the number of BCSCs by reducing IL-6 secretion. BC—breast cancer; ECM—extracellular matrix; PI3K—phosphoinositide 3-kinase; mTOR—mammalian target of rapamycin; DIRAS3—DIRAS family GTPase3; ARHI—aplasia Ras homologue member I; PFKFB3—6-phosphofructo-2-kinase/fructose-2,6-biphosphatase 3; BCSC—breast cancer stem cells; LC3—light chain 3; ALDH—aldehyde dehydrogenase; IL-6—interleukin-6; ER—estrogen receptor; HER—human epidermal growth factor receptor; TNBC—triple-negative breast cancer; STAT—signal transducer and activator of transcription; EGFR—epidermal growth factor receptor; TGFβ—transforming growth factor-β; SMAD—suppressor of mothers against decapentaplegic homolog.

Since cancer stem cells possess an ability for self-renewal and differentiate into multiple cell types, they play an important role in tumorigenesis and inducing tumor heterogeneity. BCSC was initially described as a subset of CD44^+^CD24^−^ cells, showing a characteristics of self-renewal, chemotherapy resistance, increased proliferation, and clonal mammosphere formation [87]. Further investigations discovered that a variety of phenotypes exist for BCSC such as: ALDH positive epithelial-like BCSC, CD44^+^CD24^−^ mesenchymal-like BCSC, and CD44^+^CD24^−^ALDH positive BCSC, which could transit into different phenotypes according to underlying circumstances [88,89]. A characteristic feature of BCSC is that epithelial-like BCSC is located in the center of the tumor and shows higher proliferation activity, while mesenchymal-like BCSC is in the invasive front of the tumor but is more quiescent [89]. Meanwhile, CD44^+^CD24^−^ALDH positive BCSC has the highest tumorigenic potential and invasion activity among BCSC phenotypes [89]. BCSCs are one of the factors capable of causing breast cancer metastasis [90,91]. Most early disseminated cancer cells are found in the bone marrow of breast cancer patients. These cells have a putative BCSC phenotype [92] and autophagy is implicated in BCSC cell homeostasis and cell integrity [51,90,93]. ALDH1-positive stem cells isolated from the MCF7 mammosphere have higher LC3B-related autophagy activity than those of the other groups; furthermore, BCSC proliferation and pluripotency decreased following silencing of ATG7, 4A, and BECN1 or the administration of autophagy inhibitors [90,93,94,95]. Silencing MAP1LC3 or ATG12 in HER-2-enriched breast cancer reduces the number of CD44high/CD24low BCSCs [90,96,97]. Autophagy is involved in BCSC maintenance through the IL6/STAT3, EGFR/STAT3, and TGFβ/SMAD pathways in TNBC [51,52,53,98]. This suggests that autophagy reduces the number of BCSCs by reducing IL-6 secretion. In addition, rottlerin, a natural compound, induces early autophagy through the AMPK and AKT/mTOR pathways, resulting in increased apoptosis of CD44high/CD24low BCSCs [99]. The effect of autophagy in BCSCs could differ according to the experimental condition of breast cancer (Figure 2).

## 4. Role of Autophagy in Breast Cancer Metastasis According to the Metastatic Site

The different effect of autophagy on breast cancer metastasis depends on the molecular subtype and metastatic site. The typical metastatic sites in breast cancer are the brain, bone, the lungs, and the liver [11,12,13,14,100]; however, the effect of autophagy seems to have a distinct effect considering the environmental factors; this is summarized below.

### 4.1. Brain Metastasis

Brain metastasis occurs in approximately 15–30% of late-stage breast cancer patients and is prominent in TNBC and HER-2 positive breast cancer [101]. There are two characteristics in the brain metastasis process of breast cancer: penetration of the blood–brain barrier (BBB) and crosstalk with the brain–tumor microenvironment; these characteristics are interconnected and not independent. Initially, the occurrence of brain metastasis should involve the passage of disseminated cancer cells in the circulatory system through the BBB, followed by extravasation [102,103]. The BBB is surrounded by a thick basement membrane, supported by the pericyte (fibroblast-like cells that surround endothelial cells), and connected by tight junctions. The outer BBB layer is composed of astrocytes [104]. The tight junction of the BBB is composed of claudin and occludin [105] and exhibits high electrical resistance [106]. The process of tumor cells passing through the BBB is very challenging. A representative cell encountered when breast cancer cells enter the brain is the astrocyte. An astrocyte is a glial cell that is activated when stressful conditions occur in the central nervous system and it supports the BBB [107,108]. It regulates inflammation [109], protects neurons from hypoxia [110], and provides nutrients to neurons [111]. Astrocytes promote brain metastasis of breast cancer by inducing autophagy upregulation through the CXC chemokine ligand (CXCL)12-microRNA345-KISS1 axis [112,113]. KISS1 expression inhibits ATG5/7-related autophagy to suppress breast cancer brain metastasis [112]. Its expression is significantly lower in brain metastasis than in primary breast cancer [114]. The brain uses glucose as an energy source, therefore it is in a low-glucose condition compared with other tissues. GPR94 is a glucose-regulated protein that is associated with cellular transformation and increased tumorigenesis in breast cancer. GPR94 overexpression in TNBC or HER-2-enriched breast cancer functions as a predictor of brain metastasis. GPR94 overexpression increases the resistance of brain metastatic tumor cells to the low-glucose brain environment through prosurvival autophagy [115]. Moreover, high expression of LC3B and ATG17 in brain metastasis tissues of TNBC patients is associated with poor prognosis [116].

### 4.2. Bone Metastasis

**Figure 3:**
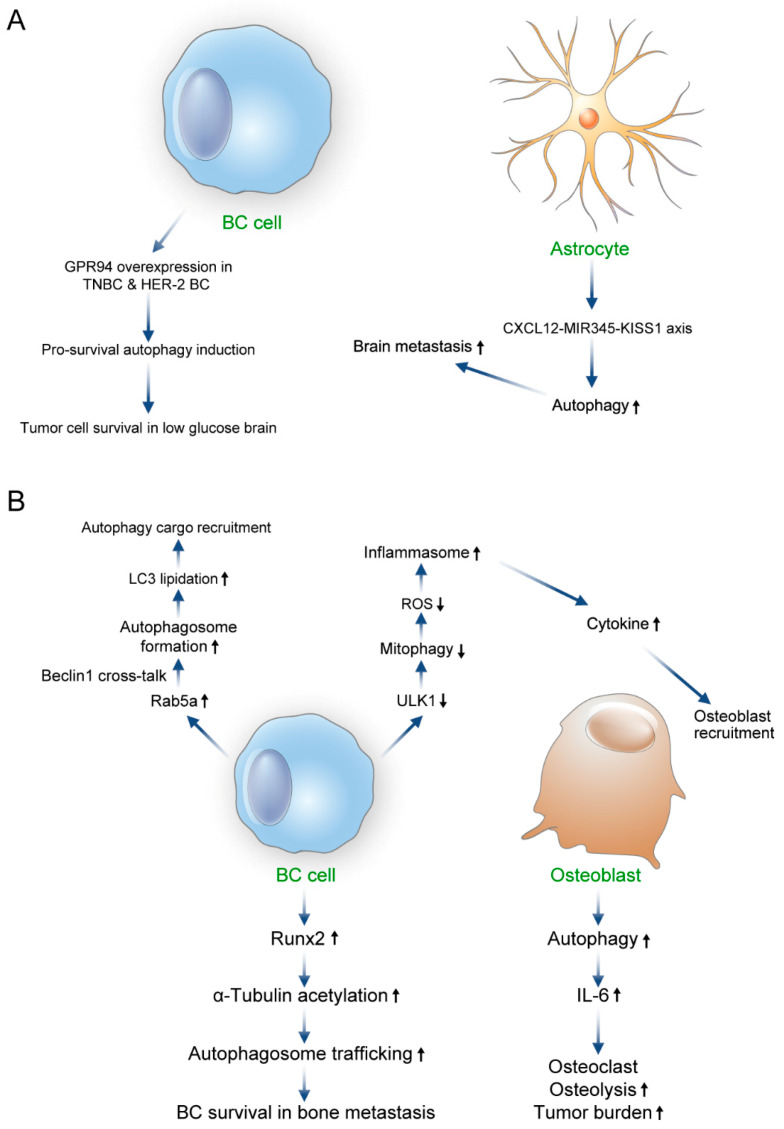
The role of autophagy in breast cancer brain and bone metastasis. (**A**) Brain metastasis of breast cancer is promoted by inducing autophagy upregulation through the CXCL12-MIR345-KISS1 axis in astrocytes, a cell present in the brain. TNBC and HER-2-positive breast cancer, which commonly metastases into the brain overexpresses GPR94; GPR94 is a glucose-regulated protein, which induces prosurvival autophagy and allows tumor cells to survive in a low-glucose brain environment. (**B**) In bone metastasis, autophagy-mediated IL-6 secretion from the osteoblast induces osteoclast differentiation and increases osteolysis and the tumor burden. Bone metastatic breast cancer cells exhibit Runx2 expression, which causes α-tubulin acetylation and increases autophagosome trafficking, thereby increasing cancer-cell survival during bone metastasis. In addition, bone metastatic breast cancer cells show an increase in Rab5a expression that promotes autophagosome formation in the early stage through crosstalk with Beclin1 and contributes to autophagy cargo recruitment by promoting LC3 lipidation in the terminal stage. ULK1 downregulation in breast cancer cells reduces mitophagy and increases reactive oxygen species (ROS) production to induce the inflammasome. This eventually results in cytokine secretion and facilitates bone metastasis through osteoblast recruitment. BC—breast cancer; GPR94—G-protein coupled receptor 94; HER—human epidermal growth factor receptor; TNBC—triple-negative breast cancer; CXCL—chemokine ligand; MIR—microRNA; LC3—light chain 3; ROS—reactive oxygen species; Runx2—Runt-related transcription factor 2; ULK—Unc-51-like kinase.

Up to 85% of patients with metastatic breast cancer have bone metastasis and approximately 60–75% of patients with metastatic breast cancer at diagnosis have bone metastasis [117]. In addition, bone metastasis shows multiple lesions in 79% of cases; the most common sites are the spine (35%), pelvis (22%), sternum (20%), and femur (8%) [118,119,120,121], demonstrating an osteolytic feature in 80% of the cases [122]. The general mechanism of metastatic bone disease is the development of bone loss by the activation of osteoclasts via pro-osteoclast factors (IL-6, receptor activator of nuclear factor kappa-Β ligand, and parathyroid hormone-related protein) secreted by tumor cells. Secretion of TGF and IGF-1 during osteolysis provokes the growth of tumor cells. The contribution of autophagy to the process of bone metastasis in breast cancer is autophagy-mediated IL-6 secretion. IL-6 can be secreted from various cells, including senescent osteoblasts. IL-6 induces osteoclast differentiation and increases osteolysis to expand the tumor burden [123,124]. In addition, Runt-related transcription factor 2 (Runx2) dysregulation is associated with bone metastasis by autophagy and Runx2 is associated with migration in breast cancer cells [125]. Runx2 expression is found in the breast cancer cell line (MDA-MB-231) and Runx2 silencing revealed that it is essential for autophagy [126]. Bone-derived breast cancer with Runx2 expression shows elevated autophagy. Runx2 triggers α-tubulin acetylation and the acetylated microtubule serves as a conduit for autophagic vesicles [126]. Acetylated microtubules are essential in autophagosome trafficking and promote autophagy [127]. Therefore, Runx2 can increase cancer cell survival during bone metastasis by increasing autophagosome trafficking through α-tubulin acetylation. Furthermore, Rab5a regulates autophagy in bone tropic MDA-MB-231 cells. Rab5a promotes autophagosome formation in the early stage of autophagy through crosstalk with Beclin1 and promotes LC3 lipidation by interacting with ATG7 in the terminal stage of autophagy. Lipidated LC3 is incorporated into the autophagosome membrane and contributes to autophagosome formation and cargo recruitment for degradation [128]. Rab5a upregulation in bone tropic MDA-MB-231 cells is over three-fold higher than in parenteral MDA-MB-231 cells. Downregulation of an autophagy kinase (ULK1) in breast cancer cells increases ROS production due to a decrease in mitophagy. Subsequently, it induces the inflammasome and cytokine secretion resulting in osteoblast recruitment and the induction of bone metastasis in a xenograft mouse model [129] (Figure 3).

## 5. Breast Cancer Metastasis Treatment using Autophagy Modulators

There are important points to consider when using autophagy as a target for tumor treatment. First, autophagy exerts tumor-promoting and tumor-suppressing effects, therefore approaches can involve either suppressing autophagy or enhancing autophagy. Second, modulation of autophagy could be conducted to stimulate current treatment methods used for tumors. Common treatment methods for tumors and tumor cells involve harsh chemotherapy or radiation therapy. The utilization of an autophagy stimulator could enhance therapeutic effects by increasing cancer cell death. Meanwhile, co-administration of an autophagy inhibitor during the occurrence of protective autophagy in the direction of inducing treatment resistance (without excessive autophagy) can alleviate treatment resistance to promote therapeutic effect. Therefore, the use of an autophagy modulator for breast cancer treatment should be considered in the context of disease stage and the previous treatment course. Modulators of autophagy that are effective in breast cancer metastasis through preclinical and/or clinical studies are stated in the following sections.

### 5.1. Autophagy Inhibitor

Chloroquine (CQ) and HCQ are well known antimalarial drugs that inhibit autophagy [130,131]. An irradiated mouse study showed that CQ significantly reduced the number of lung metastasis in a xenograft mouse study of TNBC cell lines (D2A1) [132]. Administration of HCQ with 2-deoxyglucose inhibited protective autophagy and suppressed lung and liver metastasis in a mouse xenograft study of breast cancer cells (CMT-7364 and 4T1) [133]. A clinical trial study targeting human breast cancer showed that the administration of CQ together with radiation treatment improved one-year brain metastasis progression-free survival compared with the control group (83.9% vs. 53.1%) [134] and the quinacrine antimalarial drug induces apoptosis of metastatic BCSC [135]. CQ-HF/PTX is a nanogel assembly of the chemotherapy drug paclitaxel (PTX) and amphiphilic CQ-HF polymer that inhibits excessive autophagy, inhibits paxillin degradation, and increases cell adhesion to inhibit lung metastasis of mouse breast cancer [136]. CQ and HCQ are representative autophagy inhibitors since they indirectly inhibit autophagy. They mainly inhibit palmitoyl-protein thioesterase 1, which plays an important role in tumor growth [137]. However, it is unclear whether the main effect of these drugs is due to autophagy inhibition. CD73 is an ectonucleotidase that is present on the surface of TNBC cells. Treatment with the antiCD73 antibody increases the LC3I/LC3II ratio and the p62 protein levels, while autophagy is inhibited. This inhibition significantly reduced lung metastasis in the mouse xenograft model of the TNBC cell line, 4T1 [138]. Aiduqing formula inhibits lung metastasis of breast cancer by inhibiting CXCL1-mediated autophagy [139].

### 5.2. Autophagy Inducer

Autophagy activators, such as mTOR inhibitors, can be used to treat metastatic breast cancer by enhancing cell-cycle arrest. Everolimus is an mTOR inhibitor approved for combination treatment with endocrine therapy in HR positive metastatic breast cancer [140]. The combination of everolimus and the aromatase inhibitor exemestane significantly increased overall survival in HR positive metastatic breast cancer [141,142]. Cyclin-dependent kinase (CDK)-4/6 inhibitor is an FDA-approved drug for metastatic breast cancer that suppresses the cell cycle. Inhibiting autophagy increases tumor cell death by increasing CDK-4/6 inhibitor responsiveness in cancer cells [143,144]. The co-administration of palbociclib (a CDK-4/6 inhibitor) and MLN0128 (an mTOR dual-inhibitor) reduced TNBC metastasis in a mouse model [145]. SLLN-15 is a seleno-purine molecule that induces cytostatic autophagy by inhibiting the AKT-mTOR pathway. It reduced lung metastasis of TNBC in TNBC cell lines MDA-MB-231 and 4T1 in a mouse model [146]. Pygenic acid A (PA) is a natural compound obtained from Prunella vulgaris that activates autophagy to increase LC3B I and LC3B II levels, causes p62 accumulation to sensitize metastatic breast cancer cells to anoikis, and inhibits mouse TNBC cells (4T1) and lung metastasis [129] (Table 1).

## 6. Conclusions

Autophagy plays an important role in various stages of breast cancer metastasis. Manipulating autophagy for the treatment of breast cancer metastasis has various obstacles during practical implementation. First, autophagy plays both tumor-promoting and tumor-suppressing roles in tumor biology, therefore it is difficult to determine whether suppressing or activating autophagy will be beneficial in tumor treatment. In general, autophagy contributes to the suppression of tumor development at the initial stage of tumor development and contributes to tumor progression after tumor development. Breast cancer metastasis is a multistep process, therefore a study on the role of autophagy in each step is necessary to facilitate the modulation of autophagy for the treatment of metastatic breast cancer. In general, autophagy shows a prometastatic effect in the early stage of metastasis because autophagy affects the dissemination and survival of circulating tumor cells and the maintenance and survival of dormant tumor cells. Meanwhile, autophagy shows an antimetastatic effect in the late stage of metastasis because it suppresses the emergence of aggressive cancer-cell subpopulations and macrometastatic outgrowth. In addition, it is necessary to consider how autophagy inhibition or induction will affect tumors, since autophagy has tumor-promoting and tumor-suppressing roles. For example, a prometastatic response may appear owing to a cytosolic accumulation of undigested p62 or NBR1 autophagy receptors after autophagy inhibition. Second, the function of autophagy differs depending on the breast cancer molecular subtype and metastatic organ; this should also be taken into consideration. There is a distinct difference between ER-positive luminal breast cancer and TNBC. For example, the induction of autophagy by Beclin1 suppresses tumorigenesis and tumor-cell proliferation in ER-positive luminal breast cancer, whereas autophagy is required for anchorage-dependent and independent tumor-cell growth in aggressive TNBC [67,148,149]. Furthermore, there is a distinct expression of autophagy-related proteins according to the molecular subtype of breast cancer and differences in metastatic organs according to breast cancer molecular subtypes. ER-positive luminal breast cancer has a high rate of metastasis to the bone and liver and TNBC has a greater tendency to metastases in the lung and the brain than in the other organs [13,14]. This indicates that the autophagy status may differ based on the metastasized organs. Moreover, the different microenvironment between representative organs of breast cancer metastasis (lung, bone, lung, and liver) means that there is a disparity in the tumor microenvironment that can affect the autophagy status [150,151,152]. Third, a biomarker that accurately reflects the autophagy status is required for treatments targeting autophagy. A previous study using human tumor tissues showed that autophagy-related proteins Beclin1, LC3A, LC3B, and p62 can be used indicators to reflect autophagy status. Static methods to estimate autophagy activity may not be accurate, since autophagy is a multistep dynamic process. For example, LC3 is a component of the autophagosome and higher LC3 expression can be interpreted as an increased number of autophagosomes and activity of autophagy. However, the number of autophagosomes can increase when autophagosome degradation is delayed. Therefore, it is necessary to measure autophagy flux to determine the change in autophagy steps over time to accurately measure autophagy activity [153]. However, its measurement in tissue samples has significant limitations. Collectively, developing an appropriate biomarker that properly reflects autophagy status is imperative when attempting targeted treatment of autophagy. Despite these limitations, modulating autophagy appears to have a potential in breast cancer either as a monotherapy or a combination treatment. Currently available treatments for metastatic cancer such as chemotherapy, radiotherapy, immunotherapy, and hormone therapy activate autophagy as a prosurvival mechanism. This treatment maintains tumor-cell survival; therefore, the effect of these treatments may be increased using an autophagy inhibitor. Thus, clinical trials using autophagy modulators (monotherapy or combination therapy) for metastatic breast cancer with proactive implementation and additional investigations to overcome the aforementioned limitations may justify the use of autophagy as a novel therapeutic target in the management of metastatic breast cancer.

## Figures and Tables

**Table 1:** Drug of autophagy modulator for breast cancer metastasis.

Drug	BC Type	Study Type	Results	Combined Drug or Tx	References
Autophagy inhibitor					
CQ	TNBC cell lines D2A1	Xenograft mouse study	Reduce lung metastasis	None	[132]
CQ	Human BC	Clinical trial study	Improve one-year brain metastasis progression-free survival	Radiation Tx	[134]
CQ-HF	TNBC cell lines 4T1 cell	Mouse study	Inhibit lung metastasis	Paclitaxel	[136]
HCQ	TNBC cell lines CMT-7364 and 4T1	Xenograft mouse study	Inhibit lung and liver metastasis	2-Deoxyglucose	[133]
Quinacrine	BC cell line MCF-10A-Tr	Breast cancer cell metastasis model	Induce apoptosis in metastatic BCSC	ABT-888, a PARP inhibitor	[135]
AntiCD73 antibody	TNBC cell lines 4T1 cell	Xenograft mouse study	Inhibit lung metastasis	None	[138]
Aiduqing (ADQ) formula	breast cancer cell lines MDA-MB-231, BT-549, and MCF7	Zebrafish xenotransplantation model and mouse xenografts model	Inhibit lung metastasis	None	[139]
Autophagy inducer					
Everolimus	Human BC	Clinical trial study	Increased OS in HR-positive metastatic breast cancer	Exemestane	[141,142]
MLN0128	BC cell lines (MB231, MB468, CAL148, MB453)	Xenograft mouse study	Reduce metastasis of TNBC	Palbociclib	[145]
SLLN-15	TNBC cell lines MDA-MB-231 and 4T1	Xenograft mouse study	Reduce lung metastasis of TNBC	None	[146]
Pygenic acid A	TNBC cell lines 4T1	Xenograft mouse study	Inhibit lung metastasis	None	[147]

BC—breast cancer; Tx—treatment; TNBC—triple-negative breast cancer; BCSC—breast cancer stem cells; PARP—poly ADP ribose polymerase; OS—overall survival; HR—hormone receptor.

## Data Availability

Not applicable.
